# Overview of Cannabis including Kampo Medicine and Therapy for Treatment of Dementia: A Review

**DOI:** 10.3389/fphar.2021.713228

**Published:** 2022-03-01

**Authors:** Tibor Wenger, Kazuhito Watanabe, Yui Sasaki, Keiko Kanazawa, Koichi Shimizu, Supaart Sirikantaramas, Yoshinari Shoyama, Futoshi Taura, Satoshi Morimoto, Yukihiro Shoyama

**Affiliations:** ^1^ Department of Anatomy, Histology and Embryology, Semmelweis University, Budapest, Hungary; ^2^ Daiichi University of Pharmacy, Fukuoka, Japan; ^3^ Association for Health Economics Research and Social Insurance and Welfare, Tokyo, Japan; ^4^ Molecular Crop Research Unit, Department of Biochemistry, Faculty of Science, Chulalongkorn University, Bangkok, Thailand; ^5^ Bonac Corporation, BIO Factory, Fukuoka, Japan; ^6^ Faculty of Pharmacy and Pharmaceutical Science, Toyama, Japan; ^7^ Faculty of Pharmaceutical Science, Fukuoka, Japan; ^8^ Faculty of Pharmacy, Nagasaki International University, Nagasaki, Japan

**Keywords:** *Cannabis sativa*, kampo medicine, cannabinoid biosynthetic enzyme, CB1 and CB2 receptor, anti-dementia

## Abstract

*Cannabis sativa* L. is an annual herb oldest cultivated plants as a source of fiber since about 5000 B.C. On the other hand, the cannabis flower and seed are listed in Shennong’s classic Materia Medica approximately 2000 years ago. The formulas prescribed with cannabis in Kampo medicine have been summarized. Cannabidiol (CBD) and tetrahydrocannabinol (THC) are the major neurological and psychiatric cannabinoids, and develop to drugs. It becomes evident that the therapeutic CBD and/or THC are the important candidate of anti-dementia drugs having different mechanism for Alzheimer’s patients. Two receptors and endocannabinoids are also discussed for underlying mechanism of action. In order to promote the breeding of cannabis plant containing higher concentration of target cannabinoid the biosynthetic enzymes were isolated, cloning and the tertiary structure of THCA synthase determined by x-ray analysis resulting in the possibility of molecular breeding for cannabinoids.

## Introduction

*Cannabis sativa* L. (family Cannabaceae, formerly Moraceae) is an annual herb, commonly called marijuana or cannabis in addition to many regional names (Table 1). *C. sativa* L. is used by approximately 3% of the global population as a relaxant (United Nations Office on Drugs and Crime, 2017). Tetrahydrocannabinol (THC) and cannabidiol (CBD) are the major constituents of cannabis, known as cannabinoids, and both exhibit neurological and psychiatric activities (Cohen et al., 2019; Friedman et al., 2019).

**TABLE 1:** Name of marihuana and its products in the world.

Country	Name of marihuana and its products
Algeria	Kif
Brasil	Diamba, Djamba, Liamba, Riamba, Maconha, Meconha
Edypt	Kamonga
Greek	Mavron
India	Ganja, Bhang, Charas
Jamaica	Ganga
Lebanon	Hashish el Keif
Madagascar	Rongony
Mexico	Mariguana, Marihuana, Marijuana
Morocco	Kif
Mozambique	Bangue, Suruma
Northwest Africa	Chira, Chiras
South Africa	Dagga
Syria	Hashish el Keif
Tunisia	Takrouri
Turkey	Kobak
United States	Mariguana, Marihuana, Marijuana
West indies	Mariguana, Marihuana, Marijuana

Cannabis is among the oldest cultivated plants as has been used as a source of fiber since about 5000 B.C. At present, cannabis is cultivated worldwide, as the plant is adaptable to a wide range of temperatures. The cannabis seed is considered one of five grains together with bean, corn, millet, and Japanese barnyard millet, and was first recorded in China around 1000 B.C. Three herbs, including cannabis, safflower, and indigo, together with four trees, including the tea tree, mulberry plant, lacquer plant, and paper mulberry, have been cultivated in Japan for at least 1,000 years. Among these plants, cannabis is an important source of fiber, while safflower and indigo are used to obtain dyes for the production of clothing. The cannabis flower and seed are listed among 120 safe medicinals in Shennong’s classic Materia Medica, composed during the Eastern Han Dynasty (25–220 C.E.), which lists 365 species that are divided into three categories: safe (120 items), medicinal use (120 items), and toxic substances (125 items). Therefore, cannabis can be classified as a useful plant as a source of fiber and medicine.

**FIGURE 1:**
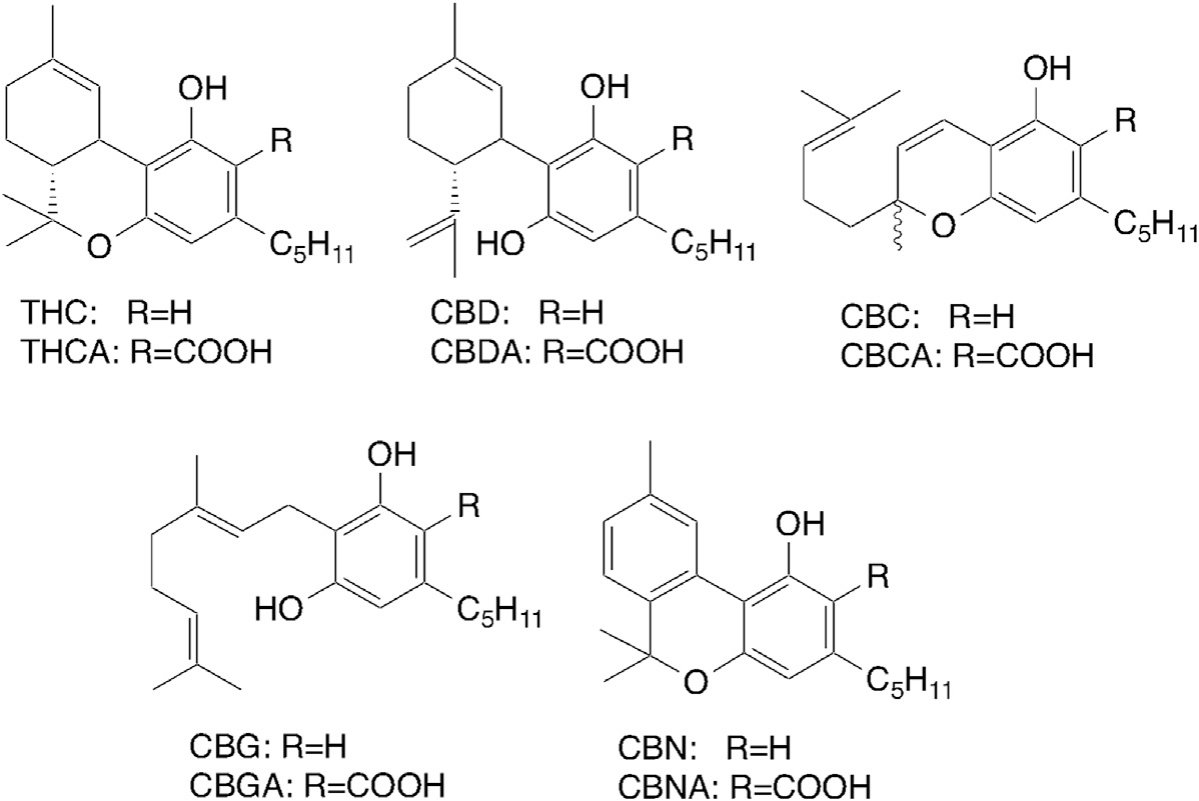
Structures of major cannabinoids.

Cannabis species contain 110 cannabinoids and 440 non-cannabinoid compounds, including terpenoids, flavonoids, and sterols (Solymosi and Köfalvi, 2017). Therapeutic studies suggest that cannabis is clinically useful for the treatment of a wide range of pathological conditions, including neurological and psychiatric disorders (Cohen et al., 2019; Friedman et al., 2019). The major cannabinoids of cannabis include CBD, THC, cannabinol, and cannabichromene (Figure 1). Adams et al. (1940) were first to describe the structure of cannabinol, while Mechoulam and Shvo (1963) and Gaoni and Mechoulam (1964) determined the structures of THC and CBD, including the positions of the double bonds and stereochemistries. Turner et al. (1980) noted the existence of 60 cannabinoids, which was increased to 70 in 2005. Elsohly et al. (2017) confirmed the isolation of 120 cannabinoids and elucidated the structures following the rapid development of analytical technologies, such as high-sensitivity mass spectrometry. It is believed that cannabinoids are sensitive against autooxidation, high temperature, lite and so on. In fact cannabinolic acid (CBNA) (Shoyama et al., 1970) and cannabicyclolic acid (CBLA) (Shoyama et al., 1972) are transformed from tetrahydrocannabinolic acid (THCA) and cannabichromenic acid (CBCA), respectively by photooxidation. However, recently Pacifici et al. (2017) confirmed that major cannabinoids, THCA and CBDA which are easily decarboxylated to give THC and CBD, respectively by heating are still stable after 1 year storage as a plant form under dark condition. This phenomenon might be occurred from the evidence that THCA and CBDA are contained in the resin composed of mainly essential oil in glandular trichomes on leaves as a sealed form (Morimoto et al., 2007).

The cannabinoids THC and CBD are the only pharmacologically active constituents of cannabis (Figure 1) and are now widely used for the treatment of chemotherapy-induced nausea and vomiting, and weight loss and intractable seizure due to HIV/AIDS.

## Kampo Medicine Prescribed With Cannabis

The cannabis flower is classified as a safe substance in the Chinese medical text Shennong Ben Cao Jing for the treatment of trauma and to activate memory. In appropriate amounts, cannabis has psychological activities, but can cause mental illness excessive amounts. Long-term intake of the seed and flower of cannabis has been proposed to maintain health. The Compendium of Materia Medica documented that the cannabis flower, called mafen, is applied as a psychotropic medication to stabilize the spirit and body to reach an immortal stage with a clear head when used in suitable amounts which might be related to anti-dementia activity as discussing in the next section. Leaves can be used for fiver and malaria. Recently Brand and Zhao (2017) introduced the mental effects of cannabis and long-term use linked to hallucinations and psychotic behaviors reported by Li Shizhen sixteen century. He Chinese surgeon Hua Tuo (140∼208 C.E.) was recorded as the first physician to use cannabis with alcohol and herbs, known as mafeisan, as an anesthetic prior to surgery. The historical details of the use of mafeisan as an anesthetic drug remain a mystery (Rafe De Crespigny, 2007). However, recently Brand and Zhao (2017) confirmed that the anesthetic prescription for decreasing paint in China was the combination of cannabis and *Datura* species flower documented in the text *Heart Text of Bian Que* (1127–1270 AD).

The cannabis seed, known as mazi in Kampo medicine, is also classified as a safe substance that can enhance visceral functions and mood. When continuously consumed for prolonged periods, body fat is reduced allowing the user to have a more youthful appearance. Kampo medicines prescribed with cannabis seeds are widely used for treatment of constipation.

*Mashiningan* is composed of the *Shojokito* formulation, which includes immature orange, magnolia bark, peony root, and rhubarb to promote excretion. The addition of cannabis seed and apricot kernel, which are oily seeds, promotes smooth and mild excretion. *Junchogan* resembles the *Mashiningan* formulation with the addition of Japanese angelica root, rehmannia root, scutellaria root, and grilled licorice, which is used to promote fluid retention and mucus secretion in the large intestine, especially in the elderly. Meanwhile, the *Junchogan* formulation is used to treat habitual constipation in the elderly with no side effects.

*Junsoto* is similar to the *Junchogan* formulation with the addition of safflower to promote the circulation of body fluids (oketsu) and has almost same pharmacological activity as the Junchogan formulation. In these formulations, anthraquinone glycosides in rhubarb promote peristalsis in the large intestine, similar to sennosides, which can transfer a sugar moiety to anthraquinone via intestinal bacteria, resulting in the production of anthracene under anaerobic conditions. Anthracene derivatives stimulate excretion by the large intestine. In this formulation, cannabis seeds promote smooth over stimulation of the intestine because of the relatively high oil content.

On the other hand, *Shakanzoto* contains cannabis seed (to moisten the intestine), ginseng, broiled licorice and cinnamon, rehmannia root, ophiopogon tuber, and donkey-hide gelatin without rhubarb, which is used for shortness of breath and palpitations with constipation-like symptoms, lack of nutrients, dry skin, and fatigue. In this formulation, cannabis seeds function to moisten the intestine and induce detoxification. Long-term use is believed to promote youthfulness and lucidness as with the *Shennong* formulation.

## Therapeutic Marijuana and Dementia

**FIGURE 2:**
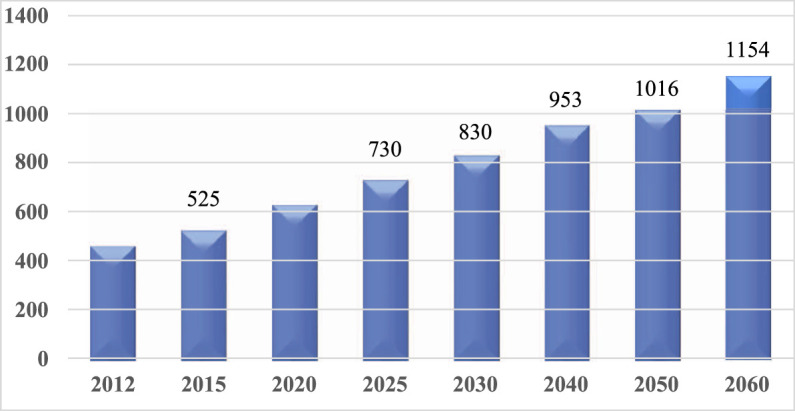
Increases of dementia patients in Japan.

The increase in human life expectancy has led to a surge in the prevalence of neurodegenerative disorders, especially dementia, worldwide. The incidence of dementia is projected to reach 81.1 million by the year 2040 (Ferri et al., 2005). In Japan, the number of dementia patients is expected to reach 7.3 million in the year 2025 and 10.2 million in 2050 (Figure 2) (National Institute of Public Health, 2015). Due to the rapid increase in the number of dementia patients, innovaventative strategies are urgently needed.

Fifth edition of the Diagnostic and Statistical Manual of Mental Disorders, the major types of dementia are Alzheimer’s disease (AD), vascular dementia, frontal lobe hyperdermia, dementia with Lewy bodies, Parkinson’s disease, Huntington’s disease, and mixed dementia. Among these disorders, AD is the most common, followed by dementia with Lewy bodies and vascular dementia. AD, which accounts for an estimated 30% of dementia cases, affects 33 million people worldwide. Typical causes of AD include neuroinflammation and oxidative stress in the brain resulting in the accumulation of amyloid-β (Aβ) plaques and tau hyperphosphorylation. It is clear that the risk of AD increases with age, but lifestyle and dietary habits are closely related to the development of dementia.

Many studies have identified various compounds with anti-dementia activities, as determined by analysis of acetylcholinesterase inhibitors (Ho et al., 2011; Natarajan et al., 2013). However, galantamine (from Galanthus caucasicus and G. woronowii) is the only natural acetylcholinesterase inhibitor currently available for clinical use. Recently, donepezil and rivistagmine have been approved for clinical use as synthetic acetylcholinesterase inhibitors, and memantine as a N-methyl-d-aspartate receptor antagonist for AD patients (Mangialasche et al., 2010). However, the side effects of acetylcholinesterase inhibitors include nausea, diarrhea, and weight loss (Kaduszkiewicz et al., 2005), while those of memantine include hallucinations, dizziness, and fatigue (Parsons et al., 1999).

THC has been approved for medicinal use by the Food and Drug Administration as a safe and effective treatment for nausea and vomiting induced by chemotherapy, and weight loss due to HIV/AIDS. THC is marketed under the names Dronabinol, Adversa, Syndros, Marinol, and Reduvo for the treatment of nausea, vomiting, weight loss, and sleep apnea. CBD under the name Epidiolex was approved in the United States and European Union in 2018 and 2019, respectively, for the treatment of intractable seizures, especially intractable epilepsy.

THC inhibits acetylcholinesterase activity and prevents aggregation of Aβ-plaques *in vitro* (Eubanks et al., 2006). On the other hand, CBD might have antioxidant activities that could affect the metabolism of anandamide, although the underlying mechanism and receptor remain unclear (Campillo and Paez, 2009; Iuvone et al., 2009; Iuvone et al., 2009). Aβ-induced neurotoxicity was protected by CBD *in vitro*. Furthermore, following challenge with Aβ proteins *in vitro*, CBD inhibited intracellular signaling pathways and suppressed tau protein hyperphosphorylation (Esposito et al., 2006a) and nitric oxide production (Esposito et al., 2006b). When the mouse hippocampus was injected with Aβ proteins, CBD induced dose-dependent suppression of the proinflammatory factors interleukin-1β and nitric oxide (Esposito et al., 2007).

Therefore, CBD may act against oxidative stress and tau phosphorylation in AD without the risk of the psychological side effects of THC.

A recent study assessed CBD for the prevention and treatment of AD (Georgia et al., 2017). An *in vivo* study conducted by Watt and Karl (2017) reported that CBD has therapeutic potential for treatment of AD. CBD exhibited anti-oxidative, neuroprotective, anti-inflammatory activities in mice injected with human Aβ proteins, while transgenic APPxPS1 mice developed Aβ plaques in the hippocampus and cortex. CBD decreased Aβ plaque-induced reactive gliosis and decreased iNOS and interleukin- 1β protein levels, resulting reduced inflammation of neuronal tissues and subsequent neurogenesis, while preventing cognitive deficits in an animal model of AD.

Moreover, administration of a combination of CBD and THC resulted in greater therapeutic activity than with CBD alone, likely due to the antagonistic response of CBD. Based on these findings, CBD is strong candidate as a prophylactic for AD because the pharmacological mechanism and efficacy are completely different than those of current drugs. Furthermore, CBD can be used clinically without the psychological side effects of THC and no concern about potential abuse.

Recently several systematic reviews have been reported. Kim et al. (2019) reviewed the publications related to the use of cannabinoid for dementia and evaluated that CBD was useful for the treatment and prevention for AD. For example CBD protects PC-12 cells against Aβ neurotoxicity and oxidation stress. Moreover, CBD inhibit acetylcholinesterase to promote memory, stimulate the neurogenesis of the hippocampus, strengthen cell survival by reducing ROS production and lipid peroxidation and so on. They concluded that the combination of CBD and THC was more effective in memory than CBD or THC alone although the psychotropic activity of THC appeared, Twelve studies were associated with inclusion criteria. Study designs were relatively scattered such as randomized controlled trials (50%) and the drug of cannabinoids, dronabinol (33%), nabilone (25%) or THC (42%). Among them dronabinol and THC were associated with significant improvements in a range of neuropsychiatric scores. The most common side effect reported was sedation. Studies under low doses of cannabinoids were evaluated to be not enough efficacy.

Although it was not proven in a randomized control trial, the observational studies showed promising results for patients having refractory symptoms. The safety evidence is good and mild. Authors suggested that dose increase and formulations having appropriated bioavailability will be needed in future (Hillen et al., 2019).

Ten female demented patients with severe behavior problems received an oral intact of higher dosages of THC and CBD likely increasing to 9.0 mg THC/18.0 mg CBD after 2 months compared to the other studies, and were well tolerated and improved behavior problems, rigidity, and daily care in severely demented patients (Broers et al., 2019).

A systematic review of randomized controlled trials reported that THC is effective against the cognitive symptoms of dementia, although evidence was less than convincing (Charernboon et al., 2021).

## Potential Benefits

Cannabis, which can be classified as a fiber, grain, oil, and/or medicine, is used by only 3% of the global population. The cannabis flower and seed have been successfully used for the treatment of psychological disorders. As mentioned above, ligands for the two types of THC receptors, CB1 (Matsuda et al., 1990) and CB2 (Munro et al., 1993) identified in the brain and macrophage, respectively, were identified as anandamide (Devane et al., 1992) and 2-arachidony glyceride (Sugiura et al., 1995). These findings have promoted the medical use of cannabinoids directly and further research of drug development from the cannabinoids THC and CBD.

Long-term use of marijuana can result in “cannabis psychosis,” which is characterized by the development of psychotomimetic and psychiatric disorders and mental instability. Also, decreased short-term potentiation and a motivational syndrome have been reported in younger people who repeatedly use marijuana (George, 2017).

However, since decriminalization policy against marihuana have been spread worldwide, the potentiality of marihuana for therapeutic use are growing louder (World drug report, 2021). In fact Brunetti et al. suggested the appropriate dosages, the analytical way of cannabinoids and the concentration of cannabinoids in marihuana prepared from different sources of herbal cannabis for prescribing doctors (Brunetti et al., 2020).

## Cannabinoid Receptors and Endocannabinoid

Two receptors and endocannabinoids will be discussed for underlying mechanism of action of cannabinoids. The ligands of two types of THC receptors, CB1 (Matsuda et al., 1990) and CB2 (Munro et al., 1993) in the brain and macrophage, respectively, were identified as arachidonoylethanolamide (anandamide) (Devane et al., 1992) and two- arachidony glyceride (Sugiura et al., 1995). It became evident that 2-arachidony glyceride is a potent ligand with high affinity for both the CB1 and CB2 receptors and the content is higher in rat brain than that of anandamide (Murielle et al., 1994). In 1998 Murielle et al. (1998) identified a selective antagonist for the CB2 receptor.

CB1 Receptor: Matsuda et al. (1990) was first to isolate, clone, and sequence a central cannabinoid receptor. Gerard et al. (1990) reported that cannabinoid receptors of the human and rat shared a 98% amino acid homology. The human cannabinoid receptor, which belongs to the seven *trans*-membrane spanning receptor family, assumes a three-dimensional conformation with 1) seven helices spanning from one side of the cell, 2) three extra-cellular and three intracellular loops, 3) a glycosylate extra-cellular N-terminal domain, and 4) an intracellular C-terminal domain involved in interactions with a G protein, which is responsible for the *trans*-membrane transduction of the receptor-mediated signal. Hua et al. (2016) elucidated the crystal structure of human CB1 and, Moldrich and Wenger (2000) used an immunohistochemical technique to identify a CB1 receptor in the rat brain and to elucidate the distribution in several other organs.

CB2 Receptor: A peripheral cannabinoid receptor, named CB2, was identified in the human spleen and was also identified as a G protein coupled 7-trans-membrane spanning receptor with 44% sequence identity with the CB1 receptor (Munro et al., 1993).

Recent advancements in endocannabinoid research have shown that most of pharmacological properties of anandamide are similar in the central nervous system (CNS) and peripheral systems with THC and other active cannabinoids, especially in regard to 1) the inhibitory effects on memory, motor activity and turning behavior (Lichtman et al., 1995; Romero et al., 1995; Terranova et al., 1995), 2) ocular blood pressure and heart rate (Varga et al., 1995; Weidenfeld et al., 1994, 3) regulation of hormones involved in the hypothalamus-pituitary-adrenal axis (Giannikou et al., 1995; Wenger et al., 1995), 4) neurotransmission mediated by dopamine, acetylcholine, noradrenalin, endorphin, glutamate, and gamma-aminobutyric acid (Wickens and Pertwee, 1993; Ishac et al., 1996, and 5) the immune response (Schuel et al., 1994; Schwarz et al., 1994). Since some of these functions are closely related to the CNS, therapeutic cannabinoids were discussed previously.

## Cannabinoid Metabolic Pathways

**FIGURE 3:**
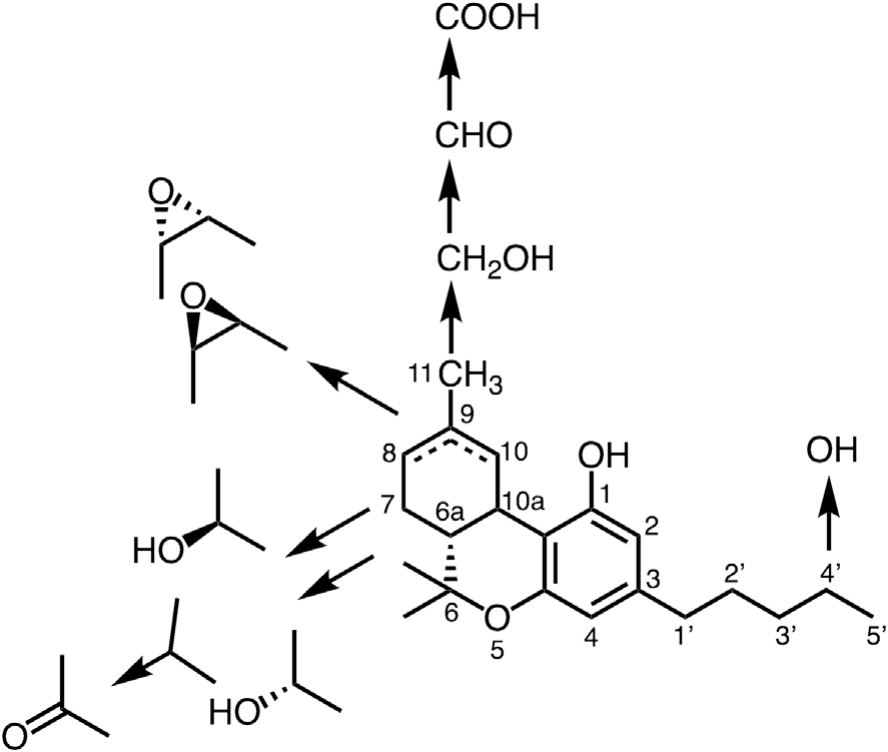
Metabolic pathway of tetrahydrocannabinol.

THC is transformed to the oxidative metabolites quickly as indicated in Figure 3 and their activities are changeable (Watanabe et al., 2000). To detect various kind of metabolites against THC a monoclonal antibody (mAb) against THCA was prepared in our lab (Goto et al., 1994; Tanaka and Shoyama, 1999). Interestingly, the anti-THCA mAb was cross-reactive against all metabolites of THC (Figure 3), CBD, and cannabinol. Furthermore, *in vitro* and *in vivo* analyses revealed rapid oxidation of THC occurred at several sites and the molecule was metabolized into hydroxyl, epoxide, aldehyde and carboxylic acid derivatives in the body. Fortunately, the mAb recognized only limited cannabinoids, but not lipophilic compounds, such as cholesterol, testosterone, β-carotene, and androstene- 3,17-dione, or the endogenous cannabinoid anandamide (Watanabe et al., 2000). An eastern blotting system will allow for one-step analysis of cannabinoid metabolites, which would be useful for pharmacological and biological analyses of cannabinoids (Shan et al., 2001). Metabolites of THC in the body are important for the bioavailability and affinity of enzymes and/or receptors. The THC metabolic pathway is shown in Figure 3 (Watanabe et al., 2005).

Elmes et al. (2015) reported a new finding related to cannabinoid metabolism that THC and CBD are carried by fatty acid-binding proteins (FABP1). Further, it becomes evident that FABP1 carries and preserves THC in ligand binding pocket temporarily, then transports to intracellular CYP450 enzymes for THC metabolism (Elmes et al., 2019) as described previously.

## Cannabinoid Biosynthesis Pathway

Recently Luo et al. (2019) succeeded to fix the biosynthetic matrix for preparation of CBGA, CBDA, THCA, tetrahydrocannabivarinic acid (CBDVA) and cannabidivarinic acid (CBDVA) (Shoyama et al., 1977) in yeast. However, since the molecular breeding for cannabinoids is needed in future, the biosynthetic enzymes of cannabinoids including isolation, cloning and the tertiary structure of THCA synthase will be discussed for further investigation based on the newly developed methodology called as missile type molecular breeding (Putalun et al., 2003; Sakamoto et al., 2012; Putalun et al., 2015).

Studies on the biosynthesis of cannabinoids started in the 1960s with the use of isotope tracers. Cannabinoids are biosynthesized via the conjugation of acetyl-malonate and mevalonate (Shoyama et al., 1994). Stout et al. (2012) confirmed that the hexanoyl-CoA formed by the acyl-activating enzyme was a precursor of cannabinoids. The conjugation of olivetorate and monoterpene moiety was occurred by a transferase to yield CBGA, the key precursor of CBDA and THCA (Fellermeier and Zenk, 1988). Further it become evident that olivetol synthase, a polyketide synthase was an essential enzyme for the biosynthesis of cannabinoids (Taura et al., 2009).

**FIGURE 4:**
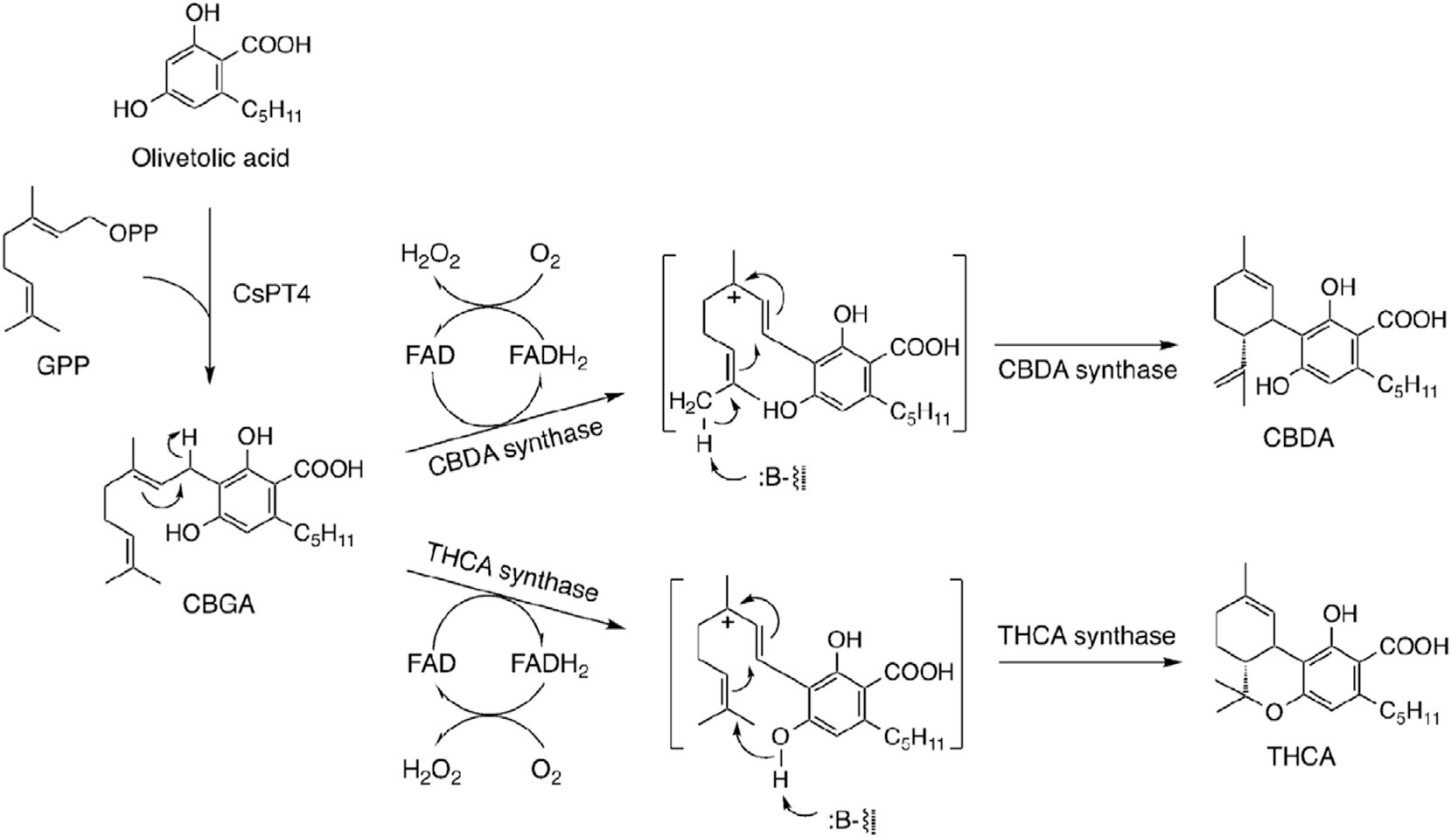
Biosynthetic pathway of CBDA and THCA [Furthermore, recently cannabinoid prenyltransferase 4 was found in *C. sativa* (Luo et al., 2019)].

Since the biosynthetic pathway was confirmed in the 1990s, three enzymes related to cannabinoid biosynthesis were isolated and purified from cannabis plants: THCA synthase (Taura et al., 1995), CBDA synthase (Taura et al., 1996), and cannabichromenic acid (CBCA) synthase (Morimoto et al., 1998). cDNA cloning of THCA synthase produces a transgenic protein (Sirikantaramas et al., 2004; Taura et al., 2007a), which is an important finding. THCA synthase is a flavoenzyme with high homology with the berberine bridge enzyme (Dittrich and Kutchan, 1991), which catalyzes oxidative cyclization of reticuline to scoulerine in the biosynthesis of benzylisoquinoline alkaloids (Zenk, 1985). These findings were confirmed by overexpression of recombinant enzymes using a baculovirus-insect cell system (Sirikantaramas et al., 2004; Sirikantaramas et al., 2005; Taura et al., 2009). CBDA synthase was also cloned and expressed as a recombinant enzyme (Taura et al., 2007b; Taura et al., 2007c). The cannabinoid biosynthetic pathway involves direct biosynthesis of THCA and CBDA from CBGA by CBDA synthase and THCA synthase, respectively (Figure 4).

**FIGURE 5:**
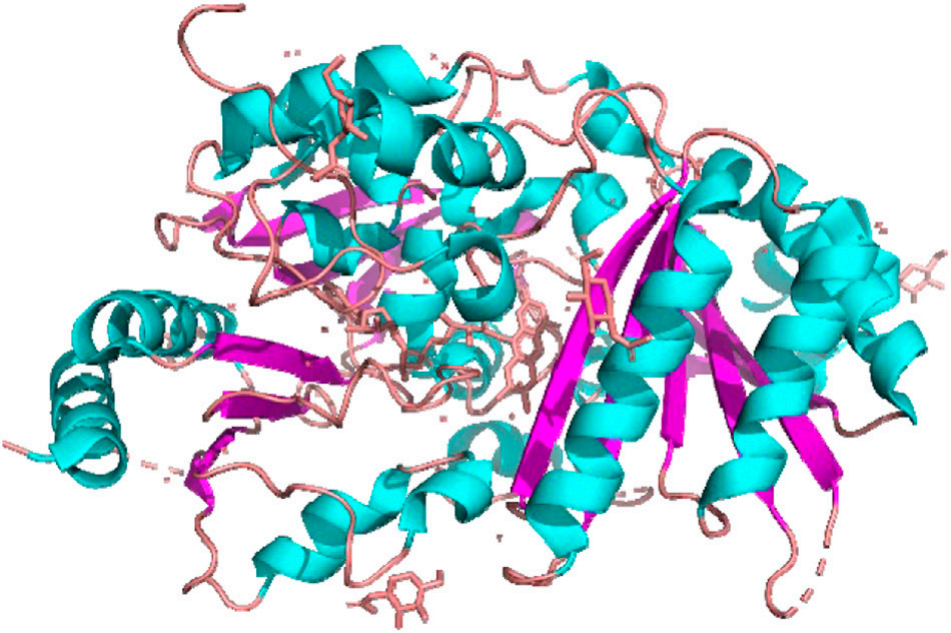
Structure of THCA synthase by X-ray crystallography.

Information on biosynthesis of cannabinoids has resulted in the crystallization and structure of THCA synthase. The enzyme was overproduced in baculovirus, and crystalized (Shoyama et al., 2005; Taguchi et al., 2008). Then, the tertiary structure was determined by X-ray crystallography at 0.75 Å resolution (Figure 5) (Shoyama et al., 2012).

The most typical characteristic of the THCA synthase molecule is a residue that binds flavin adenine dinucleotide (FAD). Based on this analysis, CBGA was identified as a substrate and THCA as a product that competed to fit into the pocket of the active site (Figure 3). Mutation experiments at the active site resulted decreased enzymatic activity of THCA synthase, but not complete inhibition. These findings suggest that the active site may function by binding with CBGA as a substrate. Therefore, the tertiary structure explains the enzymatic reaction of THCA formation from CBGA (Figure 2).

Luo et al. (2019) succeeded to set up the biosynthetic system using yeast for CBGA, CBDA, THCA, tetrahydrocannabivarinic acid (CBDVA) (Shoyama et al., 1977) and cannabidivarinic acid (CBDVA) (Shoyama et al., 1977) production without the organic synthesis and/or *Cannabis* plant. This methodology may open a new platform for preparation of natural products. Further the authors reviewed biochemistry and biotechnology on cannabinoids including new advance (Taura et al., 2019).

Cannabis can be divided into CBDA- and THCA-type strains (Kushima et al., l980). Authors group previously successfully bred a CBDA strain by the repeated crossing that mainly produced CBDA as a precursor of CBD, which was simply transformed by hort-term heating, during 5 min (Shoyama, 1993). Moreover, a mutation to THCA synthase could completely inhibit THCA synthesis and increase the CBDA content (Sirikantaramas et al., 2004). The “missile-type molecular breeding” method is a unique breeding technology in which a single chain fragment variable (scFv) gene is transformed into the host plant (CBDA strain) resulting in a three-fold increase in antigen molecules (Putalun et al., 2003; Sakamoto et al., 2012; Putalun et al., 2015). In this case, the scFv gene targeted by an anti-CBDA mAb was induced into the CBDA strain, resulting in an increased the concentration of CBDA by up to three-fold as compared to that of the original plant. These technologies strongly support the development of CBD as a drug to prevent AD as documented previously.

## Conclusion

*C. sativa* are listed as safe substances in the Chinese medical text Shennong Ben Cao Jing to maintain health and brain function at suitable dosages. The Kampo medicine such as Daiokanzoto prescribed with rhubarb and licorice can be used for the treatment of constipation in healthy persons as this formulation can cause diarrhea in the frail and elderly. Mashiningan formulation promotes milder defecation, although the high concentration of oil might be affected by the CNS to maintain mental stability without any adverse side effects.

CBD is an effective drug for the treatment of intractable seizures, especially intractable pilepsy. A recent neuronal investigation (Georgia et al., 2017) found that CBD blocked Aβ-induced neurotoxicity, tau protein hyperphosphorylation, and the activities of iNOS and interleukin-1β, highlighting a unique pharmacological mechanism as compared to currently approved anti-dementia drugs, resulting in reduced inflammation in neuronal tissues and neurogenesis for the treatment of dementia.

Two receptors, CB1 and CB2, and endocannabinoids, anandamide and 2-arachidony glyceride are discussed for underlying mechnism of action of cannabinoids in the brain. It became evident that 2-arachidony glyceride is a potent ligand with high affinity for both the CB1 and CB2 receptors. Endocannabinoid research have shown that most of pharmacological activities of anandamide are similar with THC and CBD in the CNS and peripheral systems. Metabolic pathway of THC is discussed because the metabolic speed is faster and the activity of THC decrease rapidly.

In order to make evident the biosynthetic enzyme system which has possibility for the production of higher concentration of cannabinoids, their enzymes were isolated and cloned. THCA synthase is a flavoenzyme that efficiently catalyzes oxidative cyclization of CBGA. The tertiary structure THCA synthase was determined by X-ray crystallography (Shoyama et al., 2012). This finding suggests the potential of molecular breeding of cannabis plants. For example, the cleavage of FAD from THCA synthase in the CBDA strain enhances biosynthesis of CBDA. This technology can increase the CBDA content in cannabis plants and further the development of new CBD-based drugs without the risks of psychological disorders and abuse for the prevention of dementia, especially AD.
